# Triple Pulmonary Coinfection with SARS-CoV-2, *Nocardia cyriacigeorgica*, and *Aspergillus fumigatus* Causing Necrotizing Pneumonia in an Immunomodulated Rheumatoid Arthritis Patient: Diagnostic and Therapeutic Insights

**DOI:** 10.3390/life15091336

**Published:** 2025-08-22

**Authors:** Wei-Hung Chang, Ting-Yu Hu, Li-Kuo Kuo

**Affiliations:** 1 Department of Critical Care Medicine, MacKay Memorial Hospital, Taipei 10449, Taiwan; peacejaycool@gmail.com (W.-H.C.);; 2 Department of Medicine, Mackay Medical College, New Taipei City 25245, Taiwan

**Keywords:** triple infection, necrotizing pneumonia, SARS-CoV-2, COVID-19, *Nocardia cyriacigeorgica*, *Aspergillus fumigatus*, immunosuppression, rheumatoid arthritis, ICU

## Abstract

Pulmonary coinfection involving both viral and opportunistic pathogens is an emerging challenge in immunosuppressed patients. We report the case of a 59-year-old man with rheumatoid arthritis on long-term immunosuppressive therapy who developed necrotizing pneumonia and acute respiratory failure and was ultimately diagnosed with triple pulmonary coinfection by SARS-CoV-2, *Nocardia cyriacigeorgica*, and *Aspergillus fumigatus*. Diagnosis required comprehensive imaging, bronchoscopy with BAL, and microbiological work-up. The case was complicated by septic shock, multiple organ failure, and family-driven end-of-life decisions. This report highlights the diagnostic and therapeutic complexity of triple coinfection in the ICU, emphasizing the importance of systematic microbiology, imaging, and interdisciplinary care in critically ill immunocompromised hosts.

## 1. Introduction

Triple pulmonary coinfection with SARS-CoV-2, *Nocardia cyriacigeorgica*, and *Aspergillus fumigatus* is extremely rare, especially among patients with significant immunosuppression [1,2]. Since the emergence of COVID-19, the landscape of respiratory infectious diseases has changed, leading to new challenges in both diagnosis and management in critical care settings [3,4,5]. Immunosuppressed patients—particularly those with rheumatoid arthritis (RA) on long-term corticosteroids or disease-modifying anti-rheumatic drugs (DMARDs)—are especially vulnerable to atypical and opportunistic pathogens [6,7,8]. While secondary bacterial and fungal infections are increasingly reported in patients with severe viral pneumonia, the simultaneous occurrence of triple pulmonary coinfection involving these three distinct organisms remains exceedingly uncommon but carries devastating clinical consequences [1,2,5,9].

*Nocardia cyriacigeorgica* is a Gram-positive, weakly acid-fast, soil-dwelling bacterium that mainly affects immunocompromised individuals, frequently causing necrotizing pneumonia, lung abscesses, or disseminated infections, including central nervous system involvement [7,10,11,12,13,14]. Invasive pulmonary aspergillosis, classically seen in neutropenic or profoundly immunosuppressed patients, is being increasingly identified in critically ill COVID-19 patients, especially those requiring mechanical ventilation or high-dose corticosteroid therapy [4,15,16,17,18,19,20]. The overlap between radiographic and clinical features, compounded by immunosuppression, often results in diagnostic delays and challenges in instituting appropriate therapy [16,18,20].

The current case report describes a fatal instance of necrotizing pneumonia in an immunosuppressed RA patient, complicated by the triple pulmonary coinfection mentioned above. This case emphasizes the need for a high index of suspicion, aggressive diagnostic strategies including early bronchoscopy and multiplex pathogen testing, and rapid escalation of therapy when managing pneumonia in the ICU. Additionally, it highlights the importance of multidisciplinary collaboration and ethical decision-making, especially regarding end-of-life care, in the context of overwhelming and treatment-refractory infection.

## 2. Case Presentation

### 2.1. Patient History and Initial Assessment

A 59-year-old man with a history of RA (on prednisolone 5 mg BID, hydroxychloroquine 200 mg BID, and leflunomide 20 mg QOD), hypertension, chronic kidney disease, hyperlipidemia, and a right upper ureter stone (previously treated with URSL and DJ stenting) presented to the emergency department with several days of progressive general weakness and shortness of breath. He was an active smoker (1 pack/day), unmarried, unemployed, and living alone. He was regularly followed up at the outpatient clinic for his chronic conditions.

On presentation, the patient’s vital signs revealed hypothermia (T 35.4 °C), tachycardia (HR 129/min), tachypnea (RR 22/min), and hypoxia (SpO_2_ 80% on room air, improving to 97% with 3L O_2_). He was noted to be ill-looking and confused, with crackles in the left lower lung. There was no limb edema or abdominal tenderness. Initial laboratory tests revealed leukocytosis (WBC 21,600/µL; band 3%, segmented neutrophils 86%), elevated C-reactive protein (CRP 33.1 mg/dL), procalcitonin 3.4 ng/mL, anemia (Hb 9.6 g/dL), and acute kidney injury (Cr 1.4 mg/dL, baseline ~1.0 mg/dL). Arterial blood gas showed pH 7.26, PaO_2_ 47 mmHg, and a base excess of −12.8, consistent with severe hypoxemic respiratory failure.

**Figure 1:**
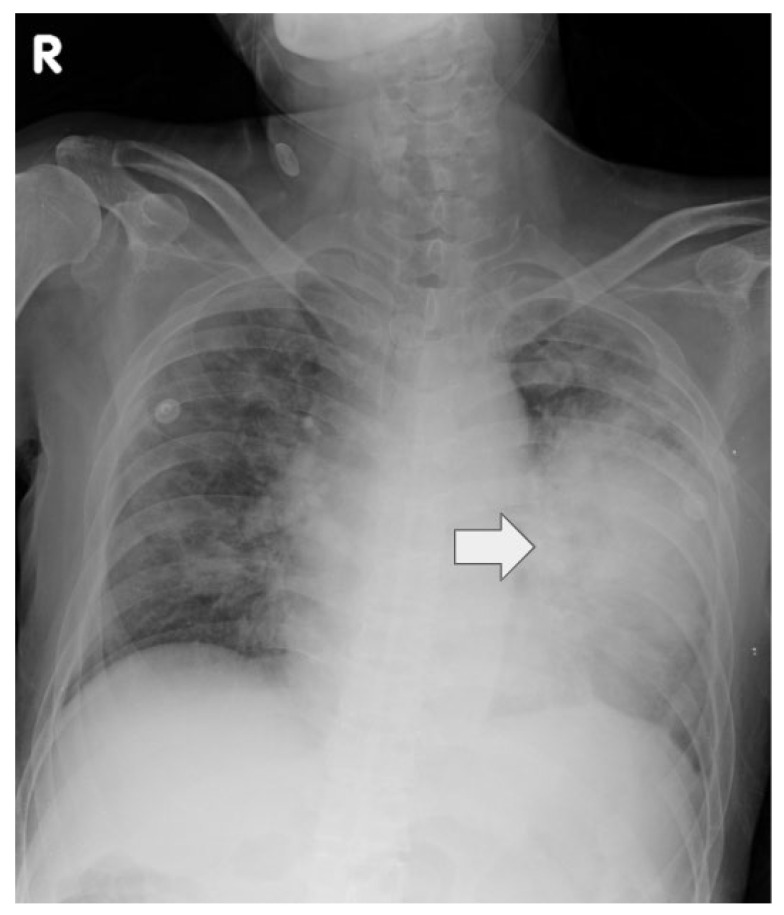
The chest X-ray obtained on admission (16 December 2023) demonstrates bilateral pulmonary infiltrates, with consolidation most prominent in the left lower lung (indicated by the arrow). The endotracheal tube is appropriately positioned. No pleural effusion is seen.

Chest radiography on admission demonstrated bilateral pneumonia with predominant left lower lobe infiltration. Given persistent and severe hypoxemia despite supplemental oxygen, the patient was emergently intubated with a 7.5 mm endotracheal tube (fixation at 22 cm) and admitted to the ICU for further management (Figure 1).

### 2.2. ICU Management, Diagnostic Work-Up, and Course

Upon ICU admission, empirical broad-spectrum antibiotics were initiated for severe community-acquired pneumonia. The patient’s immunosuppressive regimen was reviewed in consultation with rheumatology. His chronic prednisolone was tapered for adrenal replacement, and other immunomodulators were withheld.

COVID-19 testing using PCR was positive on 18 December 2023 (Ct 30.4), with a repeat positive (Ct 20.3) on 19 December 2023, confirming acute SARS-CoV-2 infection. The patient received a three-day course of remdesivir (19–21 December 2023), dexamethasone (19 December 2023), and methylprednisolone (20–23 December 2023). Prophylactic enoxaparin (18–25 December 2023) was administered. The patient was isolated in a negative-pressure room and subsequently de-isolated on 21 December 2023 after a negative rapid antigen result.

Serial chest X-rays revealed persistent bilateral consolidations. Ventilator settings were adjusted according to gas exchange and clinical response, with frequent arterial blood gas monitoring (Table 1). On 20 December 2023, bronchoscopy with bronchoalveolar lavage (BAL) was performed due to ongoing hypoxemia, copious airway secretions, and lack of clinical improvement. Bronchoscopy revealed diffuse airway inflammation and very turbid yellowish secretions, with no obvious endobronchial lesion. BAL and sputum cultures were sent for comprehensive bacterial, fungal, and viral panels.

Microbiological testing revealed the following:

- *Nocardia cyriacigeorgica* in sputum cultures (19 December 2023; 20 December 2023) and BAL (20 December 2023), confirmed by MALDI-TOF and susceptibility testing.
- *Aspergillus fumigatus* from BAL, with a positive galactomannan antigen (0.60, elevated), and positive fungal culture.
- COVID-19 PCR and antigen tests remained positive until 21 December 2023.
- Blood cultures remained negative throughout admission.
- Multiplex PCR panels for tuberculosis, *Pneumocystis jirovecii*, CMV, and HSV were negative.
- No Clostridium difficile or other major pathogens were identified in stool testing.

No antimicrobial or antifungal susceptibility data were reported for either organism. Therefore, empirical therapy with TMP-SMX and linezolid was selected for nocardiosis, and voriconazole was chosen for invasive aspergillosis, in accordance with international guidelines and expected susceptibility patterns.

No chest CT was performed due to the patient’s unstable condition and the family’s decision for palliative management. All diagnoses and monitoring were based on chest X-ray, laboratory values, and bronchoscopic findings.

Despite broad-spectrum antimicrobial therapy, including escalation to TMP-SMX and linezolid (for nocardiosis) and voriconazole (for invasive aspergillosis) after infectious disease consultation, the patient’s condition deteriorated.

### 2.3. Complications, Supportive Care, and Ethical Considerations

During the ICU stay, the patient experienced a cascade of complications:

- Septic shock (24–25 December 2023): This required the escalation of vasopressor support (norepinephrine and vasopressin). The family declined further escalation (second-line vasopressors).
- Acute kidney injury with anuria and severe hyperkalemia (up to 6.9 mmol/L): The family declined hemodialysis.
- Metabolic acidosis and persistent normocytic anemia: This required multiple packed red blood cell transfusions (19 December 2023; 24 December 2023; 25 December 2023; 28 December 2023).
- Profound hypothermia (25–27 December 2023).
- Gastrointestinal bleeding: Coffee-ground and subsequently dark red gastric aspirates were found on 27 December 2023, likely secondary to a stress-related ulcer.
- Acute pancreatitis: This was diagnosed by markedly elevated amylase (5664 U/L) and lipase (>2000 U/L) on 29 December 2023; abdominal ultrasound showed pancreatic edema and peripheral fluid accumulation.
- Transient bilateral pupil dilation without light reflex (25–27 December 2023): Neurological imaging was deferred due to the family’s preference for palliative care and avoidance of high-risk transport.
- Pressure ulcers and skin breakdown: These were managed by ICU nursing staff with standard wound care protocols.
- Ventilator management: Progressive hypoxemia required adjustments in ventilator settings, with increasing FiO_2_ and inspiratory pressure, but oxygenation continued to deteriorate.

**Figure 2:**
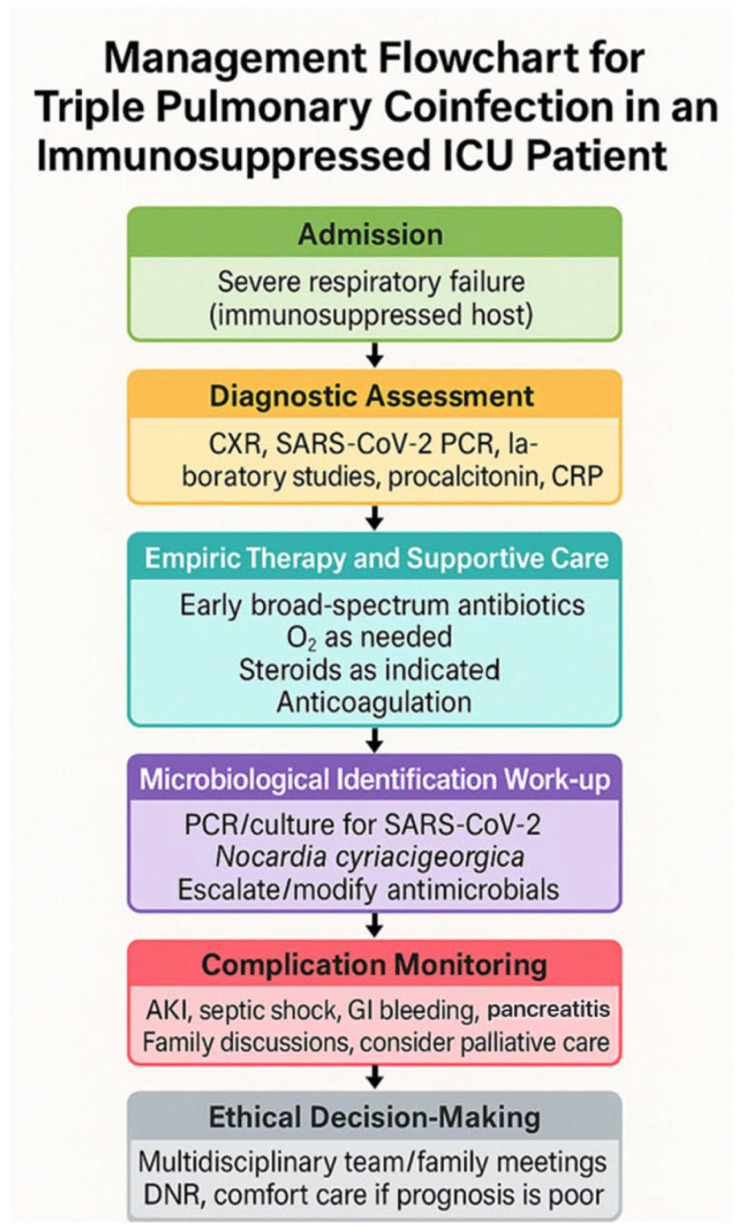
Management flowchart for an immunosuppressed ICU patient with triple pulmonary coinfection (SARS-CoV-2, *Nocardia cyriacigeorgica*, *Aspergillus fumigatus*). Key steps include diagnostic testing, empiric therapy, microbiological work-up, complication monitoring, and multidisciplinary decision-making.

After extensive family conferences, a decision was made to implement a palliative approach, including a DNR order (26 December 2023), rejection of hemodialysis, refusal of prone positioning, and a focus on comfort care. The patient expired from refractory septic shock and multiple organ failure on 31 December 2023. A summary of the ICU management pathway for this case is illustrated in Figure 2.

### 2.4. Clinical Timeline and Key Data

Table 2 summarizes the key laboratory values and their dynamic trends during ICU admission, including white blood cell count, hemoglobin, creatinine, C-reactive protein, potassium, and arterial pH, together with clinically relevant events.

## 3. Discussion

This case underscores the complexity and high mortality associated with triple pulmonary coinfection in immunosuppressed hosts [1,2,12]. *Nocardia cyriacigeorgica* typically affects patients with impaired cellular immunity, such as patients on long-term corticosteroids or with chronic kidney disease [7,10,11,12,13,14]. Clinical presentation may be subacute, with fever, cough, and progressive pneumonia, sometimes with cavitation or CNS dissemination [7,10,11]. The presence of rapidly progressive respiratory failure despite broad-spectrum antibiotics should prompt early consideration of atypical and opportunistic infections, including nocardiosis, and warrants timely bronchoscopy and BAL with comprehensive microbiological evaluation [11,18,20].

Invasive pulmonary aspergillosis, increasingly recognized in the context of severe COVID-19, is particularly associated with prolonged mechanical ventilation and corticosteroid use [4,15,16,17,18,20]. Several recent studies have highlighted the elevated risk and high mortality associated with COVID-19-associated pulmonary aspergillosis (CAPA), especially in the ICU setting [4,16,17]. Early diagnosis, based on BAL galactomannan, fungal culture, and molecular testing, is essential to guide antifungal therapy, as delayed treatment is strongly associated with poor outcomes [18,20].

In considering the possibility of colonization versus true infection, the diagnosis of invasive pulmonary aspergillosis in this patient was supported by the EORTC/MSGERC consensus criteria, including compatible host factors, radiological findings, and a positive BAL galactomannan antigen. Serum galactomannan testing was not performed, which represents an important limitation of this report and reduces the ability to definitively distinguish invasive aspergillosis from colonization. The clinical course and radiographic deterioration, along with the microbiological findings, are more consistent with invasive disease than with colonization. Additionally, other important inflammatory markers relevant to severe COVID-19 and coinfections, such as LDH, ferritin, and IL-6, were not measured in this patient. This further limits the completeness of the clinical assessment and interpretation.

COVID-19 itself is well documented to disrupt pulmonary immunity and, together with immunosuppressive therapies and critical illness, may facilitate secondary infections by organisms such as *Nocardia* and *Aspergillus* [5,21]. The clinical course is often rapidly progressive, and radiographic findings can overlap, making diagnosis particularly challenging [16,18,20].

Management of this patient required frequent adjustment of antimicrobial regimens, ventilator settings, and organ support. The escalation to TMP-SMX and linezolid for nocardiosis, voriconazole for aspergillosis, and the completion of remdesivir and corticosteroid protocols for COVID-19 exemplify the need for dynamic, evidence-based therapeutic approaches [11,18]. Nevertheless, outcomes in such cases remain poor, especially with the onset of multiple organ failure [1,2,12].

The need for family meetings and shared decision-making is highlighted in this case. The ethical complexity of care in the ICU—especially in the face of overwhelming infection and limited therapeutic benefit—necessitates ongoing dialog, transparency regarding prognosis, and, when appropriate, a shift in focus toward comfort-oriented care [22]. This case also demonstrates the importance of early recognition of palliative needs and the implementation of a multidisciplinary approach involving intensivists, infectious disease specialists, rheumatologists, and nursing staff [22].

From a diagnostic perspective, this report supports the value of comprehensive, multiplex pathogen testing (BAL, PCR, antigen, cultures) in rapidly deteriorating immunosuppressed patients [18,20]. Future directions should include the development of rapid, high-sensitivity multiplex diagnostics, as well as further research into the optimal management of coinfections in the era of COVID-19 and widespread immunosuppression [18,20].

In addition, the true incidence of triple pulmonary coinfection remains unknown, with only sporadic case reports available to date [1,2]. Previous reports on triple pulmonary coinfection involving SARS-CoV-2, Nocardia, and Aspergillus have primarily described cases in patients with hematologic malignancies or organ transplantation [1,2].

Recent studies have also reported coinfections involving SARS-CoV-2 and *Pneumocystis jirovecii*, as well as other atypical bacteria, in immunosuppressed and critically ill patients [23,24].

In contrast, our case involved a patient with rheumatoid arthritis on long-term immunosuppression, which is less commonly reported [25]. The clinical management, including the use of early bronchoscopy, broad-spectrum antimicrobials, and multidisciplinary decision-making, was similar to prior cases. However, our report highlights unique diagnostic and ethical challenges encountered in the context of autoimmune disease, as well as the importance of family-centered decision-making in end-of-life care.

In this case, antimicrobial and antifungal susceptibility data were not available; therefore, the selection of TMP-SMX and linezolid for nocardiosis, and voriconazole for invasive aspergillosis, was based on current guidelines and expected susceptibility patterns [11,18]. This lack of susceptibility data represents a limitation of our report.

This case further demonstrates the ongoing need for heightened suspicion and aggressive diagnostic strategies for opportunistic infections in non-malignant immunosuppressed populations.

## 4. Conclusions

Triple pulmonary coinfection with SARS-CoV-2, *Nocardia cyriacigeorgica,* and *Aspergillus fumigatus* is rare but can be devastating in immunosuppressed patients [1,2]. Early recognition, aggressive diagnostics, and multidisciplinary care are essential, though prognosis remains poor when organ failure develops [1,2,12]. This case emphasizes the importance of family engagement, clear communication regarding prognosis, and integration of palliative strategies when outcomes are unfavorable [22].

## Figures and Tables

**Table 1:** The timeline of key clinical events, laboratory results, and major interventions during the ICU stay of the reported patient. Key clinical events and interventions.

Date	Event/Findings	Labs/Imaging	Microbiology	Intervention
12/16	Admission, intubation	CXR: bilateral pneumonia	—	Empiric abx, ICU admit
12/18	COVID PCR+, consult	CT: necrosis	Sputum: *Nocardia cyriacigeorgica*	Remdesivir, steroids
12/19	COVID PCR+, culture	—	Sputum: *Nocardia cyriacigeorgica*	TMP-SMX, Linezolid
12/20	BAL performed	—	BAL: *Aspergillus fumigatus*	Voriconazole
12/24	Septic shock	Labs: AKI, shock	—	Vasopressors
12/27	GI bleeding	—	—	PRBC transfusion
12/29	Acute pancreatitis	Abd Sono: edema	—	Supportive
12/31	Multi-organ failure	—	—	Comfort care, expired

Abbreviations: abx, antibiotics; BAL, bronchoalveolar lavage; AKI, acute kidney injury; PRBC, packed red blood cells; Sono, sonography.

**Table 2:** Laboratory trends, including white blood cell count, hemoglobin, creatinine, C-reactive protein, potassium, and arterial pH, at clinically relevant time points during the ICU admission. Key laboratory values and trends during ICU admission.

Date	WBC (×10^3^/µL)	Hb (g/dL)	Cr (mg/dL)	CRP (mg/dL)	K^+^ (mmol/L)	pH	Comment
12/16	21.6	9.6	1.4	33.1	4.2	7.26	ICU admission
12/18	18.5	9.2	1.1	28.7	4.5	7.32	Post-intubation
12/19	17.3	8.7	1.2	24.0	4.7	7.33	PRBC 2U, Remdesivir
12/24	15.1	8.2	2.0	22.0	5.3	7.25	Septic shock onset
12/25	14.5	7.9	2.4	21.0	5.5	7.22	PRBC 1U, shock
12/28	12.8	7.8	3.5	18.4	5.8	7.18	Anuria, PRBC 2U
12/29	13.0	7.8	3.4	17.9	5.9	7.21	Acute pancreatitis
12/30	12.5	7.6	3.4	16.0	6.3	7.16	Hyperkalemia
12/31	11.8	7.2	3.4	15.7	6.9	7.10	Pre-mortem

Abbreviations: WBC, white blood cell; Hb, hemoglobin; Cr, creatinine; CRP, C-reactive protein; K^+^, potassium; PRBC, packed red blood cells; pH, arterial pH.

## Data Availability

The data presented in this study are available on request from the corresponding author.
